# Scattered genomic amplification in dedifferentiated liposarcoma

**DOI:** 10.1186/s13039-017-0325-5

**Published:** 2017-06-24

**Authors:** Nils Mandahl, Linda Magnusson, Jenny Nilsson, Björn Viklund, Elsa Arbajian, Fredrik Vult von Steyern, Anders Isaksson, Fredrik Mertens

**Affiliations:** 1 Division of Clinical Genetics, Department of Laboratory Medicine, Lund University, SE-221 84 Lund, Sweden; 2 Array and Analysis Facility, Uppsala University, Uppsala, Sweden; 3 Department of Orthopedics, Clinical Sciences, Lund University and Skåne University Hospital, Lund, Sweden

**Keywords:** Liposarcoma, Chromosomes, Amplification, 5p, 12q, Gene expression

## Abstract

**Background:**

Atypical lipomatous tumor (ALT), well differentiated liposarcoma (WDLS) and dedifferentiated liposarcoma (DDLS) are cytogenetically characterized by near-diploid karyotypes with no or few other aberrations than supernumerary ring or giant marker chromosomes, although DDLS tend to have somewhat more complex rearrangements. In contrast, pleomorphic liposarcomas (PLS) have highly aberrant and heterogeneous karyotypes. The ring and giant marker chromosomes contain discontinuous amplicons, in particular including multiple copies of the target genes *CDK4*, *HMGA2* and *MDM2* from 12q, but often also sequences from other chromosomes.

**Results:**

The present study presents a DDLS with an atypical hypertriploid karyotype without any ring or giant marker chromosomes. SNP array analyses revealed amplification of almost the entire 5p and discontinuous amplicons of 12q including the classical target genes, in particular *CDK4*. In addition, amplicons from 1q, 3q, 7p, 9p, 11q and 20q, covering from 2 to 14 Mb, were present. FISH analyses showed that sequences from 5p and 12q were scattered, separately or together, over more than 10 chromosomes of varying size. At RNA sequencing, significantly elevated expression, compared to myxoid liposarcomas, was seen for *TRIO* and *AMACR* in 5p and of *CDK4*, *HMGA2* and *MDM2* in 12q.

**Conclusions:**

The observed pattern of scattered amplification does not show the characteristics of chromothripsis, but is novel and differs from the well known cytogenetic manifestations of amplification, i.e., double minutes, homogeneously staining regions and ring chromosomes. Possible explanations for this unusual distribution of amplified sequences might be the mechanism of alternative lengthening of telomeres that is frequently active in DDLS and events associated with telomere crisis.

**Electronic supplementary material:**

The online version of this article (doi:10.1186/s13039-017-0325-5) contains supplementary material, which is available to authorized users.

## Background

Cytogenetic analyses of more than 3200 benign and malignant soft tissue tumors have revealed that different patterns of chromosomal aberrations exist among these lesions [1, 2]. Several tumor entities are characterized by specific, sometimes pathognomonic, structural rearrangements, mostly translocations, giving rise to oncogenic fusion genes, often with no or few other changes of chromosome number or morphology. Another set of tumors displays a moderate number of chromosomal imbalances, whereas still another set of tumors shows highly complex karyotypic rearrangements with extensive cytogenetic heterogeneity. Both losses and gains of sequences may be of pathogenetic importance. While losses affect one or both copies of one or more genes, gains can range from one to hundreds of extra gene copies. Moderate and high level gene amplification manifest cytogenetically as intrachromosomal homogeneously staining regions (hsr), extrachromosomal double minutes (dmin) or ring chromosomes (r); other mechanisms behind amplification are presumed to be rare and are not easily recognized by chromosome banding analysis. Among soft tissue tumors, ring chromosomes are much more abundant than dmin, and hsr is even more infrequent (Additional file 1). Ring chromosomes, allowing for gene amplification through breakage-fusion-bridge cycles [3], constitute the characteristic cytogenetic feature of some soft tissue tumors, including atypical lipomatous tumor/well differentiated liposarcoma (ALT/WDLS) and dedifferentiated liposarcoma (DDLS).

Modern array technologies have revealed that gene amplification is more common among neoplastic cells than detected by banding analyses. Such technologies, however, do not reveal the chromosomal organization of multiplied sequences, which might provide some clues about how they originated and their evolutionary potential. In the present study, amplification through scattering over many chromosomes is described in a case of dedifferentiated liposarcoma.

## Methods

As part of a study of soft tissue sarcomas that at G-banding analysis showed aberrations including add(5)(p15), FISH analyses were performed in order to find out if the breakpoint in 5p was localized to a restricted position that could indicate the involvement of a particular gene. No consistent pattern was found, but one case showed a peculiar distribution of chromosome 5 sequences, which prompted further investigation.

The patient was a 67-year-old man with a deep-seated tumor in the left thigh. The largest diameter of the highly necrotic, infiltratively growing tumor was 24 cm. Two samples – an open biopsy and the resected specimen – were obtained with an interval of five weeks. The diagnosis was dedifferentiated liposarcoma with atypical fat cells, sclerosis, a spindle cell component, as well as a component of spindle cells with rhabdoid differentiation; no region compatible with a well-differentiated liposarcoma was seen. Postoperative radiation therapy was given. X-ray two years later revealed no apparent lung metastases. Five years after diagnosis, metastases to the lungs and soft tissues on the back appeared. The patient died soon after.

Chromosome preparations were made from short-term cultured cells obtained from disaggregated tumor tissue from both samples and stained for G-banding as previously described [4].

FISH analyses were performed using whole chromosome painting probes wcp5 (green) and wcp12 (blue) (Vysis, Downers Grove, IL). Site-specific probes were CTD-2074D8 (5p14.1–14.3), RP11-509B9 (5p15.1), RP11-35 K22 (5p15.32), CTD-3080P12 (5p15.33), hereafter referred to as D8, B9, K22 and P12, respectively, as well as RP11–1137 N1 (12q14.3–15) for detection of the *MDM2* gene (BACPAC Resource Center; https://bacpacresources.org). The following fluorophores were used for labelling: red, Cy3 dUTP (VWR), green, Chromatide Alexa Fluor 488–5-dUTP (Thermo Fisher Scientific). Hybridizations were performed as described [5]. No material was available for further analyses.

SNP array analysis of the two samples was performed as described [6]. In brief, tumor DNA (250 ng) was extracted and analyzed using the Affymetrix CytoScan HD array (Affymetrix, Santa Clara, CA, USA). Genomic aberrations were identified by visual inspection using the Chromosome Analysis Suite version 1.2 (Affymetrix). The human reference sequence used for alignment was the GRCh37/hg19 assembly. Constitutional copy number variations were excluded through comparison with the Database of Genomic Variants (http://dgv.tcag.ca/dgv/app/home). Further bioinformatic analysis regarding copy numbers and segmentation was performed using Rawcopy and the Tumor Aberration Prediction Suite (TAPS), as described [7, 8]. Since the chromosome number was at the triploid level, only copy numbers of at least 6 were considered true amplification. Mean and median copy numbers were calculated as well as the total length of amplified sequences.

RNA sequencing (RNA-Seq) was performed on the excised tumor biopsy, as described [9]. Identification of potential fusion transcripts was performed on fastq files using FusionCatcher [10]. The GRCh37/hg19 build was used as the human reference genome. Expression of some selected candidate target genes in 5p and known targets in 12q was compared with their expression in a set of myxoid liposarcomas.

## Results

**Fig. 1:**
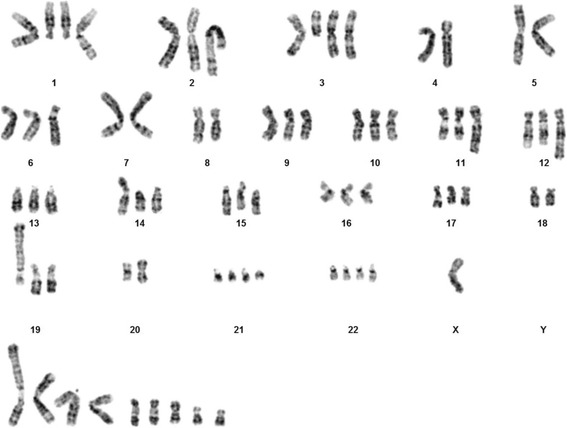
G-band karyogram showing fairly complex chromosomal aberrations

A hypertriploid, complex karyotype was found in both samples (Fig. 1). The only difference between the two samples was a slight variation in chromosome number, 70–74 and 73–76, respectively. Based on both samples the composite karyotype was interpreted as 70–76,XX,-Y, +1,del(1)(q12)×2,add(2)(p1?),+del(3)(q11),-4,-5,add(5)(p15),?add(5)(p11),-7,add(7)(p11)×2,-8,-10, −11,?add(11)(q22),?ins(12;?)(q13;?)×2,der(12)add(12)(p11)add(12)(q24),add(14)(p11),add(19) (q13)×2,?der(19)add(19)(p11)del(19)(q12),-20,+21,+22,inc[cp24].

**Fig. 2:**
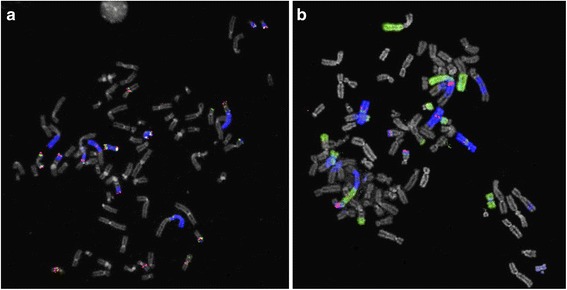
Metaphase FISH images showing multiple signals (**a**) for 5p site-specific probes (*green and red*) and wcp5 (*blue*), and (**b**) for *MDM2* (*red*) as well as wcp5 (*green*) and wcp12 (*blue*)

**Fig. 3:**
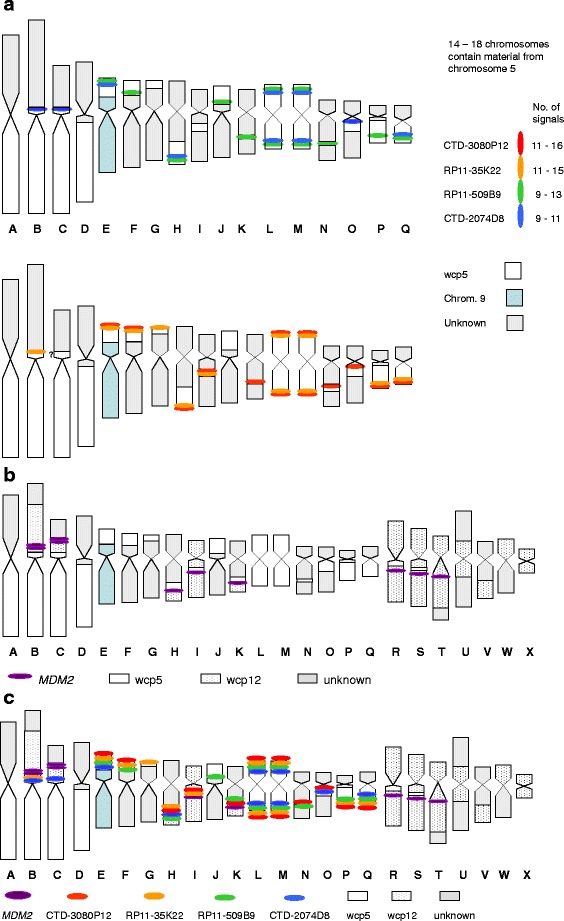
Schematic representations of the FISH results. **a** analysis using four site-specific 5p probes and wcp5; **b** analysis using a probe detecting *MDM2* as well as wcp5 and wcp12; **c** summary of all FISH analyses. Letters A to X are used as identification of different aberrant chromosomes

FISH analyses revealed that chromosome 5 sequences were spread to several chromosomes (Fig. 2). Using wcp5, no intact chromosome 5 was found, but wcp5-positive segments were detected in 14–19 chromosomes, at least 17 of which were clonal (Fig. 3a). Large segments were seen in four chromosomes (designated A-D in Fig. 3a) and three of these most likely contained the centromere of chromosome 5. Two identical chromosomes (L and M) could represent i(5)(p10). One chromosome (E) was identified, based on the DAPI staining pattern, as a derivative chromosome 9 with chromosome 5 material added to the truncated 9p. FISH using the more proximal probes D8 and B9 revealed 9–11 and 9–13 signals, respectively, per metaphase. The corresponding number of signals for K22 and P12 were 11–15 and 11–16 respectively. Similar signal patterns for all four probes of the two probe sets were seen in chromosomes E, H, L, M, and Q.

Chromosome 12 sequences were identified in 12 chromosomes (Fig. 3b). In most metaphases there were 10 signals for *MDM2* located in eight chromosomes. Sequences from both chromosomes 5 and 12 were present in seven chromosomes (B, C, H, I, K, R, and S); in R and S no signals for the site-specific 5p probes were detected. Twelve chromosomes were positive for wcp5 but not for wcp12 (A, D-G, J, and L-Q), whereas five chromosomes were wcp5 negative but wcp12 positive (T-X). A summary of several FISH analyses is shown in Fig. 3c.

**Fig. 4:**
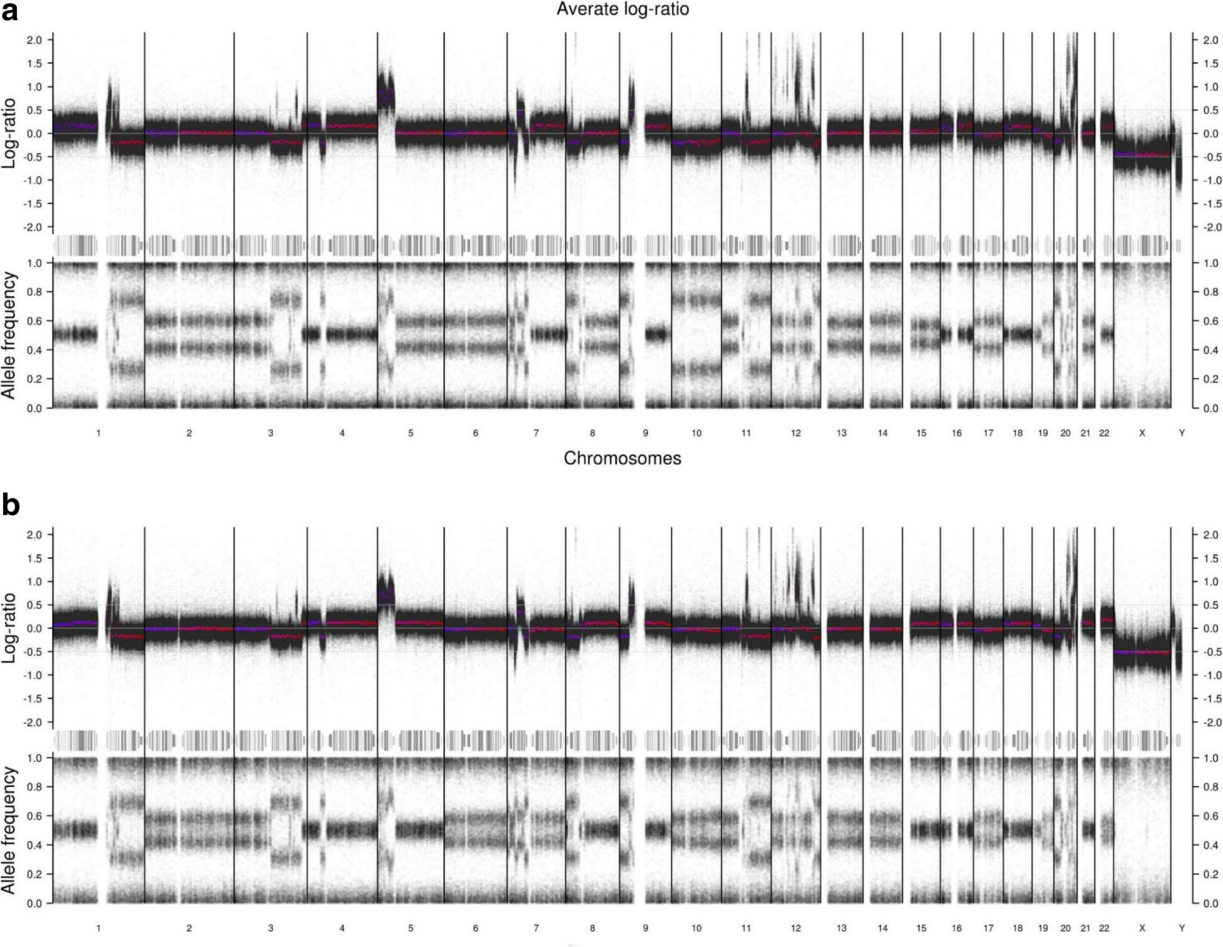
Log ratio and B-allele frequency from SNP array profiles of **a** the first and **b** the second sample of the DDLS. The log ratio was normalized to a near-triploid karyotype. Thus, log ratio 0.0 represents 3 copies and in the first sample 4 and 2 copies have log ratios 0.2 and −0.2, respectively. The corresponding shifts in allele frequencies (AF) could be exemplified by chromosome arms 1p (2 maternal +2 paternal copies; AF 0.5), most of 1q (2 + 0 copies; AF 0.75), and chromosome 2 (2 + 1 copies; AF 0.6) in the first sample. Both copy number and AF profiles are less distinct in the second sample, presumably due to larger fraction of stromal cells

At SNP array analysis, amplified sequences were found on chromosome arms 1q, 3q, 5p, 7p, 9p, 11q, 12q, and 20q (Table 1, Additional file 2). Few differences were found between the two samples (Fig. 4). The chromosome 5 amplicons emanated from almost the entire short arm, with peak copy numbers from p15.33-p15.32, p15.31-p15.2, p14.3, and p14.1-p12. The major parts of 5q were estimated to 4 copies. In chromosome 12, discontinuous high level amplicons were found from q12 to q24.21. There were about 10 copies of sequences covering the *HMGA2* and *MDM2* genes, whereas *CDK4* was estimated to 17 copies. In general, amplified sequences in 12q showed higher copy numbers than those in 5p. The size of increased copy numbers in 5p and 12q corresponded to 46.3 Mb and 17.2 Mb representing about 94% and 17% of the length of these chromosome arms, respectively. Gain/amplification in other chromosomes was found for sequences within 1q21.2-q22 and 1q24.1, 3q26.2, 7p15.2-p12.3, 9p21.3-p13.1, 11q13.2-q13.4 and 11q22.1, and 20q11.23-q13.33, representing about 7%, 2%, 23%, 32%, 5% and 37%, respectively, of the chromosome arms. Among these chromosomes, only chromosome 20 displayed more extensive high level amplification (12 copies), in particular confined to 20q13.2-q13.33.

**Table 1:** Distribution and size of chromosome segments showing amplification

Chromosome arm	Mean copy number	Median copy number	Extension (Mb)	Fraction of arm with amplification
1q	8.1	8	8.462	6.8%
3q	7.2	7	2.013	1.9%
5p	9.9	10	46.273	93.6%
7p	6.8	6	13.772	23.2%
9p	8.1	8	13.905	31.5%
11q	12.0	9	3.866	4.8%
12q	12.4	11	17.159	17.4%
20q	13.3	12.5	13.212	36.6%
Total			118.662	

Only copy numbers of at least 6 are included

**Fig. 5:**
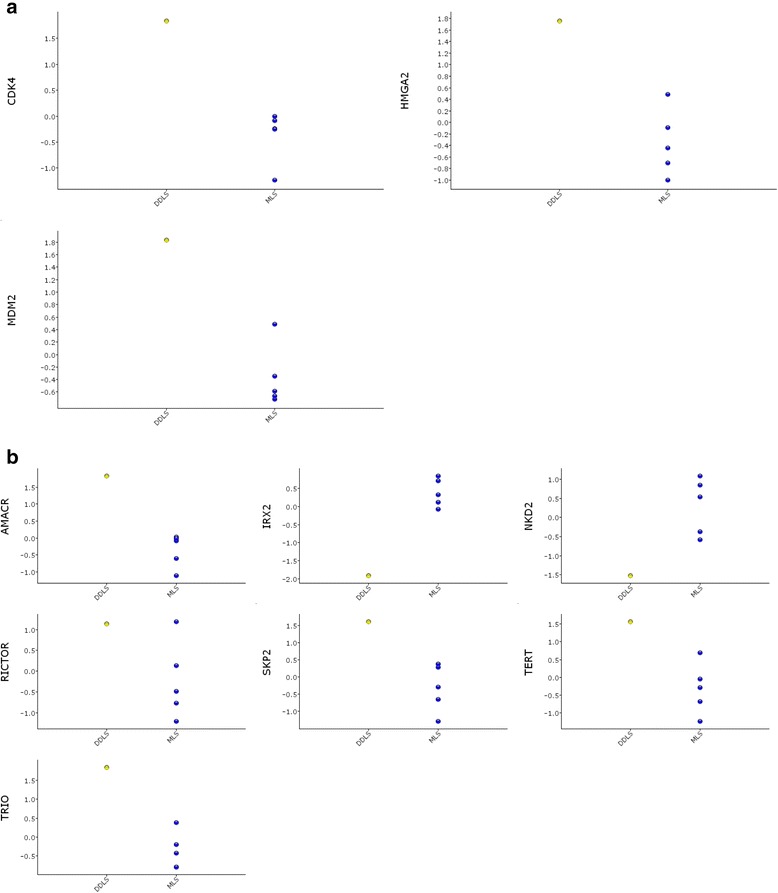
Scatter plots of the expression of some selected genes in **a** 12q and **b** 5p in the present case of DDLS compared with 5 samples of myxoid liposarcoma (MLS)

None of the potential fusion transcripts that were detected at RNA-Seq was considered significant (Additional file 3). Of the selected target genes, *AMACR* and *TRIO* in 5p and *CDK4*, *HMGA2* and *MDM2* in 12q showed significantly (*p* < 0.05) elevated expression in relation to myxoid liposarcomas (Fig. 5).

## Discussion

ALT/WDLS share many cytogenetic characteristics with DDLS - supernumerary ring chromosomes and/or giant marker chromosomes constitute the hallmark of the reported karyotypes from ALT/WDLS (*n* = 174) and DDLS (*n* = 27). On average, DDLS tend to have somewhat more complex karyotypes than ALT/WDLS, whereas the 15 published karyotypes of pleomorphic liposarcomas (PLS) are distinctly more complex and ring chromosomes are much less frequent [2] (Additional file 1). Similarly, near-diploid stemline chromosome numbers predominate in ALT/WDLS and DDLS, but are rare in PLS. The ring and giant marker chromosomes in ALT/WDLS and DDLS always contain sequences from 12q, typically with several separate amplicons that invariably include *MDM2* (and often also *CDK4* and *HMGA2*), and frequently also segments from one or more other chromosomes [11–17]. Available data show that sequences from almost all chromosome arms may be co-amplified with 12q; the only exceptions - Yp, Yq and the p-arms of the acrocentric chromosomes - could be due to the methods of detection. The most commonly involved chromosome arms are 1q (46%), 6q (22%), 7p, 8q, 9q (13%), 1p, 4p, 14q (12%), 5p, 12p, 20q (11%) and 16q (10%). The non-random co-amplification of certain regions suggests that they harbour genes of potential pathogenetic significance, or that they contain sequences prone to recombine with 12q amplicons.

DDLS usually also show more copy number changes than ALT/WDLS [16]. A comparison between the well-differentiated and dedifferentiated components of the same tumor revealed more aberrations among the latter, but no particular sequence(s) could account for the dedifferentiation process [12, 18]. Even more extensive genomic reorganization is found among PLS. The dominating (>15% of cases) amplifications include sequences from 1q and 12q in WDLS, from 1p, 1q, 6q, 8q and 12q in DDLS, and from 5p and 20q in PLS (Table 2). A conspicuous difference is the paucity of 12q amplification in PLS. A clear trend of increasing frequencies in WDLS to DDLS to PLS is seen for amplifications in 5p and 20q – 3%, 13%, 23% and 0%, 6%, 23%, respectively. Such a trend is not seen for amplifications in any other chromosome arm. Possibly, these differences indicate that some gene(s) in 5p and 20q are of importance for tumor aggressiveness.

**Table 2:** Amplification of sequences from chromosome arms in WDLS, DDLS and PLS, based on literature data

Chromosome arm	Ampl. WDLS (%) *n* = 79	Ampl. DDLS (%) *n* = 32	Ampl. PLS (%) *n* = 22	Chromo-some arm	Ampl. WDLS (%) *n* = 79	Ampl. DDLS (%) *n* = 32	Ampl. PLS (%) *n* = 22
1p	0	19	9	11q	0	6	0
1q	30	28	9	12p	3	6	0
2p	3	0	0	12q	76	88	0
2q	0	9	0	13q	0	9	5
3p	0	3	0	14q	4	13	5
3q	3	3	5	15q	0	9	0
4p	5	9	0	16p	0	0	0
4q	3	3	0	16q	0	3	0
5p	3	13	23	17p	0	0	5
5q	0	0	0	17q	0	0	0
6p	0	6	0	18p	1	0	0
6q	5	31	14	18q	3	3	0
7p	3	9	5	19p	1	3	0
7q	1	3	0	19q	4	9	0
8p	4	6	0	20p	0	3	5
8q	4	16	0	20q	0	6	23
9p	0	3	5	21q	4	0	5
9q	3	9	0	22q	0	0	5
10p	0	9	0	Xp	0	3	0
10q	0	0	0	Xq	0	0	0
11p	1	9	0	Yp, Yq	0	0	0

Amplification of 5p segments is not confined to adipocytic tumors, but has been reported in other soft tissue sarcomas, as well as in epithelial neoplasms. Among sarcomas, it is preferentially seen in tumors typically characterized by complex chromosomal aberrations, such as myxofibrosarcomas, undifferentiated pleomorphic sarcomas, leiomyosarcomas and angiosarcomas, some of which are difficult to distinguish from PLS [19–28]. Also some other non-mesenchymal tumors, such as urinary bladder cancer, non-small cell lung cancer, cervical cancer and multiple myeloma, show 5p amplification with amplicons to some extent overlapping those found in sarcomas (e.g., [29–32]). These data further support the suggestion that amplification of genes in 5p may be associated with aggressive tumor growth. Information on concomitant amplification of 5p and 12q sequences is only available in some of the tumor types listed above, but data indicate that it is not common among PLS, leiomyosarcoma, or myxofibrosarcoma. Findings from array analyses support the paucity of extra copies of both 5p and 12q in PLS; it is rare in WDLS, but found in about one-fourth of DDLS (Table 3).

**Table 3:** Fraction of borderline and malignant adipose tissue tumors with copy number changes in 5p and 12q13–21

Chromosome segment^a^		WDLS	DDLS	PLS
5p	12q13–21			
0	0	0.01	0	0.14
0	G	0.21	0.09	0.09
0	A	0.73	0.63	0
G	0	0	0.03	0.36
A	0	0	0	0.23
G	G	0.01	0	0.18
G	A	0.01	0.13	0
A	A	0.03	0.13	0
Summary of the figures above, making no distinction between gain and amplification				
0	G/A	0.94	0.72	0.09
G/A	0	0	0.03	0.59
G/A	G/A	0.05	0.26	0.18

These calculations are based on available literature data

^a^0 = no copy number change; G = gain; A = amplification; G/A = gain or amplification

Apart from amplified 12q sequences, regularly confined to ring and giant marker chromosomes, the chromosomal distribution of other amplified sequences is less well documented. Co-amplified chromosomal material, in particular from 1q, has, however, been shown to be intermingled with 12q sequences in rings and giant markers [13, 33–35]. The present case, showing both 5p and 12q amplicons, fits well with a minor subset of DDLS, but is atypical in the sense that it displays a near-triploid chromosome complement without any ring or giant marker chromosomes. Moreover, the complex pattern of amplification of 5p and 12q sequences, together in the same chromosomes and separately in different chromosomes, is unusual. Admittedly, there is no definition of what should be regarded as a giant marker, but those described in the literature are typically at least twice as large as chromosome 1. The size of the largest aberrant chromosome (B) containing 5p and 12q sequences in the present case was 1.5 times the length of chromosome 1, as estimated from G-banding (Fig. 3c). The vast majority of the 24 chromosomes with wcp5 and/or wcp12 signals were much smaller than chromosome 1. Only rare cases of ALT/WDLS with amplification in medium-sized linear chromosomes have been reported [36, 37]. No similar pattern of amplified sequences scattered over so many chromosomes has been reported before.

Genes located in 5p that have been suggested as possible amplicon targets in sarcomas include *NKD2*, *TERT*, *IRX2*, *TRIO*, *AMACR*, *SKP2* and *RICTOR* (e.g., [24, 38–40]). In the present case, these genes were amplified at similar levels (about 10 copies), but with a slightly lower level for *IRX2* and a slightly higher level for *TRIO*. On average, the amplification levels of sequences covering almost the entire 5p were lower than the levels seen in 12q and 20q, In particular, one of the well documented targets in 12q, *CDK4*, was highly amplified (about 17 copies). Also, in 5p there were very few and short intervening sequences showing a copy number corresponding to the ploidy level, in contrast to 12q and 20q where such intervening sequences were more abundant and mostly much larger, resulting in a more discontinuous amplicon pattern. This could indicate that the gene gains in 5p are less important, or merely passengers, or that 5p contains no or few genes that, if amplified, would counteract cell survival or proliferative advantages conferred by the amplified target genes. Negatively acting genes, from the tumor cell’s perspective, would be selected against. Also neutral passenger genes would gradually be lost since they represent a replication cost affecting the tumor cells’ fitness. However, it is not necessarily so that higher copy numbers are a sign of pathogenetic impact. First, the copy number is not always directly correlated with expression at the protein level [41]. Second, the tuning of protein co-expression is delicate. Amplified genes can affect the activity of many non-amplified genes and too many copies of some genes could be counterproductive for the cancer cell fitness.

The origin of the observed scattered pattern of amplified 5p and 12q sequences is obscure. Most probably, there was an early rearrangement involving substantial parts of chromosomes 5 and 12 resulting in a mitotically unstable, possibly dicentric, structure. Through further structural reorganization, *MDM2*/*CDK4* and 5p genes may have been positioned close to each other and then spread to other chromosomes, sometimes together and sometimes separately. The many chromosomes involved indicate a stage of karyotypic instability, which may have been transient. Although the observed karyotype may represent a sideline in the tumor cell population that was preferentially dividing in vitro, or biased sampling, it did not, despite its complexity, show signs of extensive heterogeneity. A possible initial event could be chromothripsis, a phenomenon that in itself does not result in copy number changes, but can be a starting point [42, 43]. However, some of the aberrant chromosomes, in particular B but also C, containing large segments of both chromosomes 5 and 12 are hardly compatible with amplification following chromothripsis. An alternative scenario might be related to the mechanism of telomere length maintenance active in the tumor. Instead of activation of the telomerase-associated mechanism many sarcomas use alternative lengthening of telomere mechanisms. This is rare in WDLS and myxoid liposarcomas, but fairly common in DDLS and the dominating mechanism in PLS [44]. Part of the alternative lengthening of telomeres confers a destabilization of the genome through nuclear receptor binding to telomeres resulting in multiple inserted interstitial telomere sequences that are fragile and thus recombination prone [45]. This mechanism alone or, more likely, combined with an early mitotically unstable structure including 5p and 12q sequences as alluded to above could lead to amplification and a spreading of these sequences to a variety of chromosomes. Indeed, other mechanisms may be responsible for the observed pattern of scattered amplification. Recent studies have demonstrated that telomere crisis and telomere healing can have dramatic and multiple effects on the genome [46, 47]. These include polyploidization as well as chromosome instability that may lead to kataegis or chromothripsis-like aberrations.

Whether the present tumor represents an exceptional case remains unknown since few similar studies of liposarcomas without ring or giant marker chromosomes have been reported.

## Conclusions

The finding of genomic amplification through distribution of 5p and 12q sequences, together and separately, to many chromosomes in a DDLS lesion represents a novel cytogenetic pattern of copy number gains. This contrasts with amplification through formation of ring or giant marker chromosomes commonly seen in WDLS and DDLS. The amplicons of 12q were discontinuous, whereas those of 5p comprised almost the entire arm. Apart from *CDK4*, *HMGA2* and *MDM2* in 12q, candidate target genes in 5p contributing to pathogenesis include *TRIO* and *AMACR* that showed elevated expression.

## Additional files

Additional file 1: Fraction of lesions with cytogenetically detectable structures associated with gene amplification among soft tissue tumors. (DOC 104 kb)

Additional file 2: SNP array results from the two tumor samples. (ZIP 720 kb)

Additional file 3: Putative fusion transcripts detected at mRNA sequencing. (XLSX 26 kb)
