# Supplementary material for: Scattered genomic amplification in dedifferentiated liposarcoma
Source: Mol Cytogenet. 2017 Jun 24;10:25. doi: 10.1186/s13039-017-0325-5 (PMC5483303; doi:10.1186/s13039-017-0325-5)
Supplement: Supplementary file 1 — Fraction of lesions with cytogenetically detectable structures associated with gene amplification among soft tissue tumors. (DOC 104 kb) [file 13039_2017_325_MOESM1_ESM.doc]

Additional file 1. Fraction of lesions with cytogenetically detectable structures associated with gene amplification among soft tissue tumors. Only entities where more than five cases with abnormal karyotypes have been reported are included^a^.

| Tumor entity | No. of cases | r (%) | dmin (%) | hsr (%) |
| --- | --- | --- | --- | --- |
| Lipoma | 480 | 4.2 | 0 | 0 |
| Lipoblastoma | 64 | 10.92 | 0 | 1.6 |
| Chondroidlipoma | 6 | 33.3 | 16.7 | 0 |
| Angiomyolipoma | 8 | 0 | 0 | 0 |
| Spindle cell / pleomorphic lipoma | 28 | 0 | 0 | 0 |
| Hibernoma | 19 | 5.3 | 0 | 0 |
| Atypical lipomatous tumor | 174 | 84.5 | 4.6 | 0.6 |
| Dedifferentiated liposarcoma | 27 | 88.9 | 18.5 | 3.7 |
| Myxoid / round cell liposarcoma | 119 | 5.0 | 0.8 | 0 |
| Pleomorphic liposarcoma | 15 | 26.7 | 20.0 | 0 |
| Liposarcoma NOS | 19 | 36.8 | 15.8 | 10.5 |
| Adipocytic tumor special type | 21 | 4.8 | 0 | 0 |
| Fibroma | 8 | 0 | 0 | 0 |
| Desmoplastic fibroblastoma | 11 | 0 | 0 | 0 |
| Angiofibroma | 10 | 0 | 0 | 0 |
| Solitary fibrous tumor | 63 | 1.6 | 0 | 3.2 |
| Superficial fibromatosis | 64 | 0 | 0 | 0 |
| Desmoid-type fibromatosis | 51 | 3.9 | 0 | 0 |
| Myofibroblastic sarcoma | 24 | 37.5 | 4.2 | 0 |
| Dermatofibrosarcoma protuberans | 47 | 66.0 | 0 | 0 |
| Fibrosarcoma | 43 | 2.3 | 0 | 0 |
| Low-grade fibromyxoid sarcoma | 25 | 28.0 | 0 | 0 |
| Myxoinflammatory fibroblastic sarc | 11 | 36.4 | 9.1 | 0 |
| Myxofibrosarcoma | 73 | 20.5 | 6.8 | 5.5 |
| Fibro-/myofibroblast tumor special type | 15 | 6.7 | 0 | 0 |
| Localized giant cell tumor | 19 | 10.5 | 0 | 0 |
| Diffuse-type giant cell tumor | 28 | 0 | 0 | 0 |
| Benign fibrous histiocytoma | 17 | 11.8 | 0 | 0 |
| Leiomyoma | 494 | 6.9 | 0.2 | 2.4 |
| Myogenic sarcoma | 11 | 9.1 | 45.5 | 0 |
| Leiomyosarcoma | 132 | 18.2 | 10.6 | 10.6 |
| Rhabdomyosarcoma NOS | 18 | 0 | 0 | 0 |
| Rhabdomyosarcoma embryonal | 82 | 2.4 | 11.0 | 1.2 |
| Rhabdomyosarcoma alveolar | 101 | 2.0 | 15.8 | 1.0 |
| Rhabdomyosarcoma pleomorphic | 10 | 20.0 | 10.0 | 0 |
| Muscle tumor special type | 20 | 30.0 | 0 | 0 |
| Pericytoma | 6 | 0 | 0 | 0 |
| Epithelioid hemangioendothelioma | 7 | 28.6 | 0 | 0 |
| Kaposi sarcoma | 6 | 0 | 0 | 0 |
| Angiosarcoma | 13 | 7.7 | 0 | 0 |
| Soft tissue tumor NOS | 13 | 15.4 | 7.7 | 7.7 |
| Myxoma | 29 | 0 | 0 | 0 |
| Angiomatoid fibrous histiocytoma | 7 | 14.3 | 0 | 0 |
| Neurofibroma | 12 | 16.7 | 8.3 | 0 |
| Schwannoma | 87 | 3.5 | 0 | 1.1 |
| Perineurioma | 9 | 0 | 0 | 0 |
| Mal peripheral nerve sheath tumor | 124 | 16.9 | 8.1 | 1.6 |
| Ossifying fibromyxoid tumor | 7 | 0 | 0 | 0 |
| Myoepithelioma/myoep carc/mixed | 24 | 16.7 | 4.2 | 0 |
| Synovial sarcoma | 228 | 5.3 | 0.4 | 0 |
| Epithelioid sarcoma | 17 | 5.9 | 5.9 | 0 |
| Alveolar soft part sarcoma | 12 | 0 | 0 | 0 |
| Clear cell sarcoma | 40 | 2.5 | 2.5 | 0 |
| Desmoplastic round cell tumor | 13 | 0 | 0 | 0 |
| Undifferentiated sarcoma | 9 | 11.1 | 0 | 0 |
| Undifferentiated pleomorphic sarcoma | 110 | 35.5 | 12.7 | 0.9 |
| Undifferentiated round cell sarcoma | 11 | 0 | 0 | 0 |
| Soft tissue tumor special type | 32 | 21.9 | 3.1 | 0 |

^a^ Data extracted from the Mitelman Database, version January 2017 [2].
